# Systemic Immune Response to Traumatic CNS Injuries—Are Extracellular Vesicles the Missing Link?

**DOI:** 10.3389/fimmu.2019.02723

**Published:** 2019-11-20

**Authors:** Abi G. Yates, Daniel C. Anthony, Marc J. Ruitenberg, Yvonne Couch

**Affiliations:** ^1^Department of Pharmacology, Medical Sciences Division, University of Oxford, Oxford, United Kingdom; ^2^School of Biomedical Sciences, Faculty of Medicine, University of Queensland, Brisbane, QLD, Australia; ^3^Acute Stroke Programme, RDM-Investigative Medicine, University of Oxford, Oxford, United Kingdom

**Keywords:** extracellular vesicles, traumatic brain injury, spinal cord injury, inflammation, acute phase response

## Abstract

Inflammation following traumatic injury to the central nervous system (CNS) persists long after the primary insult and is known to exacerbate cell death and worsen functional outcomes. Therapeutic interventions targeting this inflammation have been unsuccessful, which has been attributed to poor bioavailability owing to the presence of blood-CNS barrier. Recent studies have shown that the magnitude of the CNS inflammatory response is dependent on systemic inflammatory events. The acute phase response (APR) to CNS injury presents an alternative strategy to modulating the secondary phase of injury. However, the communication pathways between the CNS and the periphery remain poorly understood. Extracellular vesicles (EVs) are membrane bound nanoparticles that are regulators of intercellular communication. They are shed from cells of the CNS including microglia, astrocytes, neurons and endothelial cells, and are able to cross the blood-CNS barrier, thus providing an attractive candidate for initiating the APR after acute CNS injury. The purpose of this review is to summarize the current evidence that EVs play a critical role in the APR following CNS injuries.

## Introduction

Acute CNS injuries, including traumatic brain and spinal cord injury (TBI; SCI), as well as stroke, are a major global burden (1, 2). These neurological disorders have a collective global incidence rate of 500–700 per 100,000 people (3), and have extremely high morbidity, requiring lifelong subsequent care at a substantial financial and emotional cost (4, 5). Whilst the primary causes of TBI and SCI, and even to some extent stroke, are largely unavoidable, the ensuing secondary injury and ongoing inflammatory response can significantly worsen outcome and could be amenable to therapeutic intervention (6–9). The mechanisms that promote the inflammatory response to injury, and the communication pathways that convey messages about CNS health status to the systemic immune system, are the subject of intense investigation, but it is becoming clear that extracellular vesicles (EVs) play a pivotal role.

### Acute CNS Injury—Primary vs. Secondary Injury

Damage to the CNS following a neurotraumatic event occurs in two distinct phases (7, 10, 11). The primary phase is largely mechanical, whereby the physical insult causes direct structural damage to neuronal tissue and the vasculature, resulting in immediate cell death, and hemorrhage, ischemia and/or oedema.

The primary phase occurs within a short window of time, whereas the secondary phase has been shown to persist for days, weeks, even months after the injury (12, 13). Although not damaged directly during the initial insult, CNS tissue surrounding the injury is highly vulnerable to secondary damage (10, 14). Hypoxia, excitotoxicity, free radical formation, breakdown of blood-CNS barriers and release of proteases, all contribute to further cell death (10, 15). Moreover, activated microglia and astrocytes, as well as infiltrating leukocytes from the periphery, release cytokines and chemokines that create a pro-inflammatory microenvironment (6, 7, 13). Collectively, this results in the progressive destruction of CNS tissue, known as “bystander tissue damage”, which considerably impairs functional recovery (16).

Previous studies utilizing rodent models have shown that the secondary phase of traumatic CNS injury is dependent on the acute phase response (APR), a systemic inflammatory response occurring predominantly in the liver (17). In response to CNS damage, hepatic expression of pro-inflammatory mediators significantly increases as early as 2 h post-insult (17–21). In turn, these mediators trigger the mobilization and priming of leukocytes from the bone marrow, which then translocate to the site of injury, as well as seemingly uninvolved peripheral organs. The spleen releases its reservoir of pro-inflammatory monocytes and increases expression of IFN-γ, TNF, and IL-6 amongst others (22–24). Systemic inflammatory response syndrome (SIRS) which can lead to multi-organ dysfunction syndrome (MODS) is also not uncommon in patients (25–29). Concurrent immunosuppression of the adaptive immune components is often observed (30, 31), leaving patients also highly susceptible to infections. Peripheral immune responses thus significantly increase patient mortality and morbidity.

Interestingly, suppression of the peripheral inflammatory response has been shown to ameliorate CNS inflammation (20, 32–35). Modulation of the APR by targeting the production of acute phase proteins, or Kupffer cell depletion, both reduce neutrophil recruitment to the CNS in models of TBI and SCI (20, 33). Therefore, suppression of the APR may offer an alternative strategy of minimizing tissue loss and functional deficits after traumatic CNS injuries. However, it must be acknowledged that modulating systemic inflammation is complex; paradoxically, exacerbating periphery inflammation has similarly been shown to reduce lesion size and leukocyte infiltration of the CNS post-injury (36, 37). As such, it has been suggested that the systemic response can also serve as an immune “distraction”, redistributing leukocyte populations from the injured CNS to other sites, although it remains unclear to where the leukocytes redistribute (17). It is likely that timing of the inflammatory insult is key, and improving our understanding of it will ease therapeutic targeting.

The initiation signal for the activation of the peripheral response is unclear. Both humoral and neuronal methods have been investigated, yet vagotomized animals still exhibit an APR (38, 39), and thus far no consistent molecular candidates have been identified that can fully explain this response (40). There is growing evidence that extracellular vesicles, novel mediators of communication between distant organs, provide the missing link.

### Extracellular Vesicles

**Figure 1:**
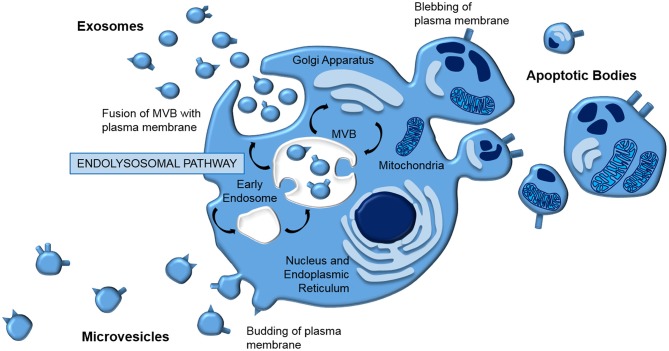
EV biogenesis. EVs are typically classed according to their biogenesis. Apoptotic bodies and microvesicles are released from the plasma membrane in blebbing and budding mechanisms, respectively. In contrast, exosomes are generated by the endolysomal pathway; internal budding of an endosome results in a multivesicular body which fuses with the plasma membrane, releasing exosomes by exocytosis.

Extracellular vesicles (EVs) is a general term that defines all cell-derived particles encapsulated in a lipid bilayer, which are enriched for proteins, lipids, and nucleic acids (41–44). They are typically classified according to their biogenesis (Figure 1); apoptotic bodies (1,000–5,000 nm) are released from the plasma membrane as part of programmed cell death, microvesicles (150–1,000 nm) are blebbed from the cell membrane, whilst exosomes (40–150 nm) are generated via the endolysosomal pathway and stored in multivesicular bodies (MVB) prior to release by exocytosis.

Whilst EVs have been investigated as a phenomenon for more than 30 years, the significant role EVs play in intercellular communication is only just being recognized. Indeed, a plethora of studies have identified EVs as important mediators of not only normal physiology, but also of pathology. They have been shown to be released from almost all cell types, including neurons (45, 46), microglia (47, 48), astrocytes (35), and CNS endothelial cells (49). EVs have also been isolated from almost all bodily fluids, including cerebrospinal fluid (CSF) (50, 51) and plasma (52). They have shown a unique capacity to disseminate information around the body, including across the blood-CNS barrier (35), to exert their effects both locally and systemically to distant organs, making them attractive candidate mediators of CNS-to-immune communication following injury.

EV-mediated cell communication has been associated in a number of neurological diseases, where they have been shown to be vectors of pathogenic proteins, propagating both Alzheimer's and Parkinson's disease (53–56). In brain cancers, EVs derived from tumor cells have been shown to act locally in facilitating proliferation, growth and angiogenesis (57–60), as well as distally in other organs aiding metastasis (61). In turn, distal cancers are able to metastasize to the brain via EVs as well (61–63). In the periphery, circulating EVs isolated from LPS-treated animals have been shown to induce gliosis and expression of pro-inflammatory molecules in the brains of naïve mice (64). Moreover, EVs released from stimulated brain endothelial cells have been shown to induce hepatic TNF and CXCL1 expression in naïve rats, in turn inducing a sickness behavior phenotype (49). Together, these studies suggest the presence of a CNS-periphery communicatory axis that is mediated by EVs. As such, investigating EVs in the context of traumatic CNS injuries is of great interest. Here, we will evaluate the current evidence that EVs mediate the communicatory pathways between the CNS and the periphery following traumatic CNS injury.

## Traumatic Brain Injury (TBI)

TBI is a devastating disorder, affecting over 55 million people globally (2). The current lack of available treatments is commonly attributed to gaps in our knowledge of the secondary phase of injury (14). Human clinical data has confirmed that TBI induces a robust inflammatory response in the periphery which is predictive of poor outcome (65). In conjunction, numerous studies have consistently demonstrated that circulating EVs are significantly elevated in TBI patients during the acute phase of injury (66, 67). For example, Nekludov et al. (66) showed a transcranial gradient in EV concentration; more EVs were detected in the cerebrovenous compared to arterial blood, indicating that the increase in circulating EVs originated from the brain. Increases in EVs in the circulation of patients with TBI have been reflected in rodent studies (35, 48, 49, 52, 68, 69). Hazelton et al. (52) showed an increase in plasma EVs during the first 24 h after TBI, whilst Couch et al. (49) and Dickens et al. (35) both showed increases in an IL-1β model of inflammatory focal brain lesions. Critically, inhibition of EV release from the CNS has been shown to attenuate the systemic response to brain inflammation, and subsequently inhibit leukocyte infiltration (35). Nekludov et al. further demonstrated that whilst leukocyte- and platelet-derived EVs were increased after injury, the circulating EVs were predominantly of endothelial origin, the concentration of which was 7-fold greater than in healthy controls. Dickens et al. (35) showed however that a proportion of plasma EVs released after striatal IL-1β injection are derived from astrocytes, and that these translocate to the liver, spleen, and lung, further linking EV-mediated signaling with the APR following CNS injury. Microglia and astrocytes both release EVs in response to DAMP-mediated activation with ATP (47). In turn, microglia-derived EVs enriched for IL-1β have been reported in the plasma of TBI patients (48). From these studies, it is easy to assume that EV population changes are due to increased release from cells of the CNS. However, EVs derived from hematopoietic cells can also signal to the brain, and their uptake here was exacerbated by peripheral inflammation (70). Delineating the origin of EVs could identify the critical players in CNS-periphery communication, and may identify a specific cellular target for EV-based therapeutics.

Functional analysis of plasma EVs from models of brain injury determined that plasma EVs were pro-inflammatory and able to induce a systemic inflammatory response in naïve rats, in the absence of CNS injury (49). It has been established that EVs are capable of interacting with granulocytes and lymphocytes; they have been shown to carry MHC class I and II, and contribute to antigen presentation (71–75). Therefore, they may directly activate the peripheral immune system through receptor-ligand mechanisms. Moreover, microvesicles and apoptotic bodies are enriched for phosphatidylserine (PS) on the outer leaflet, which not only assists in promoting budding, but also encourages uptake by macrophages and dendritic cells (76, 77). This is highly relevant considering the ongoing apoptosis of CNS cells post-injury. Kumar et al. (48) showed that EVs depleted of their content with the surfactant PEG-TB had lost their ability to activate microglia *in vitro*, making it clear that the composition of EVs is vital for them to exert their effect.

As well as surface chemistry, EV cargo appears to be key to the function of the EVs after TBI. Plasma EVs isolated from TBI patients were found to have distinct and unique protein profiles in comparison to those isolated from healthy controls (68, 78). When exogenous pro-inflammatory EVs were administered intravenously to a model of TBI, the EVs were found to exacerbate both the APR, and the subsequent neuroinflammation and pathology (52). Importantly, this response was dependent on the cellular origin of the EVs. Particles derived from macrophages had the greatest effect on hepatic expression of pro-inflammatory molecules, as well as infiltrating neutrophils in the brain, compared to those from endothelial cells and plasma samples. Cargo analysis revealed differential miRNA content in the different EVs, suggesting the particles exert their effect through transfer of specific genetic transcripts. Indeed, Dickens et al. (35) identified that miRNA in astrocyte-derived EVs target the PPAR-α pathway, leading to increased NFκB activity and cytokine production in the liver. However, EVs have been found to be enriched for pro-inflammatory molecules themselves, including cytokines, chemokines, and inflammasome proteins. Inflammatory EVs have been reported to transport IL-1β (47, 48, 79, 80), IL-6 and CCL2 (81), as well as chemokine receptors, such as CCR5 (82). Collectively, these studies suggest a more direct mechanism of initiating and propagating inflammation.

In addition to the activation of a systemic inflammatory response, TBI-associated coagulopathy (TBI-AC) has been associated with EV signaling (83). Following injury, TBI patients often develop a hypercoagulable state, leading to an increased risk of thrombosis (84–86). This has been associated with increased mortality (84), and platelet dysfunction has been reported to play a causal role (83). It is thought that the circulating EV population is predominantly shed from platelets (87), and these platelet-derived particles have greater procoagulant activity than platelets themselves (88). TBI induces the release of EVs from platelets (66, 67, 69), and circulating microparticles following TBI were shown to have procoagulant properties *ex vivo* (89). Moreover, Tian et al. (69) were able to reproduce systemic coagulopathy in uninjured mice through adoptive transfer of TBI plasma EVs. Together, these data indicate that platelet-derived EVs may be responsible for TBI-AC, which could be attributed to the exposure of PS on the outer EV leaflet. It is also likely that brain-derived particles interact with platelets directly to promote systemic coagulation and thrombosis. Astrocyte- and neuronal-derived EVs have been isolated from the blood of TBI animals, and were found to be procoagulant in phenotype (69). Thus, EV-mediated changes in systemic function are not limited to alterations in inflammatory status after injury.

## Spinal Cord Injury (SCI)

In comparison to brain pathologies, the role of EVs following SCI has been somewhat overlooked. Whilst systemic inflammation has been well-documented in SCI patients (26, 90–93), studies have focused on its contribution to functional outcome rather than the manner in which it is communicated. To our knowledge, there is currently no data that describes changes in the circulating EV population and their influence on pathophysiology of SCI. That being said, EVs have been isolated from the CSF of deceased SCI patients (94). These EVs were found to be enriched for the inflammasome-associated proteins NLRP1, caspase-1, and ASC, suggesting a pro-inflammatory phenotype. The authors speculated that these EVs may be able to trigger an innate immune response *in vivo*, which would correspond with TBI associated data, however, EV-mediated effects on systemic inflammation and immune activation were not investigated. These authors additionally demonstrated that neuronal exosomes loaded with siRNA could localize to the lesion epicenter following SCI when injected systemically, further supporting the hypothesis of an EV-mediated CNS-periphery communicatory axis.

Preliminary, unpublished data from our group suggest that SCI induces a significant increase in plasma-derived EVs during the acute phase of injury, which is consistent with human and animal models with brain injuries. However, it is necessary to determine the specific role of these SCI-induced changes in the circulating EV population in propagating peripheral inflammation and the subsequent effect on lesion development. Whilst TBI data may provide some insight, it must be acknowledged that the overall impact on the APR and lesion progression is likely to be different (17). Anatomically, the distribution of gray and white matter, as well as the distribution and phenotype of microglia are quite different in the spinal cord compared to the brain. Moreover, they both respond differently to traumatic injury in that the blood-spinal cord barrier (BSCB) shows greater breakdown after trauma compared to the blood-brain barrier (BBB), and also that there is increased local CXC chemokine expression and recruitment of neutrophils to the parenchyma of the spinal cord compared to the brain. Regarding the systemic response, peripheral administration of the PPARα agonist fenofibrate blocked the APR and neutrophil recruitment to the brain after an intrastriatal microinjection of IL-1β injection (35), however it was found to be an ineffective treatment in experimental SCI (95). These differences must be taken into consideration when assessing the impact of EV signaling following injury, as manipulation of the cascade after SCI may have differential effects on lesion progression and patient recovery compared to TBI.

## EVs as Therapy

It is clear that interrupting EV signaling may be useful to treat inflammation, but some groups have also used the EVs themselves as a therapeutic agent, specifically EVs derived from stem cells. This strategy is certainly attractive, circumventing the ethical issues with embryonic and fetal stem cells, as well as being less invasive with low or no tumorigenicity. Moreover, the ability to use autografted stem cells will eliminate the risk of rejection. Most studies to date have almost exclusively utilized EVs released by mesenchymal stem cells (MSCs), and these have consistently been shown to improve functional recovery and behavior deficits in models of TBI (96, 97) and SCI (98–100). EVs derived from progenitor cells, such as endothelial colony-forming cells (101) and neural stem cells (102), appear to have similar neuroprotective effects in animal models. Kobayashi et al. (103) demonstrated that EVs derived from induced pluripotent stem cells (iPSCs) were able to both increase angiogenesis and the rate of wound closure in a model of skin wound healing. Whether iPSC-EVs have therapeutic potential in the context of TBI/SCI remains to be investigated.

The mechanisms underlying the neuroprotective actions of stem cell-derived EVs are currently under investigation. To date, they have been shown to be internalized by endothelial cells (101), neurons (104), astrocytes (104), oligodendrocytes (105), and microglia (106) in the CNS, suggesting they may exert their effect directly. However, improvements after injury are not necessarily due to prevention of cell death, as no change in lesion volume has often been reported (97, 107). Rather, EVs may exert their effect by stimulating endogenous restorative mechanisms that promote recovery. Zhang et al. (97) have shown MSC-EVs enhanced vascular density and neurogenesis, with a concurrent reduction in brain inflammation in a TBI model. Increased angiogenesis has also been shown in a model of SCI (108), following treatment with MSC-EVs. One potential mechanism that has been proposed is the transfer of miRNAs (11). Xin et al. (104) demonstrated that EV-associated miR-133b transferred to astrocytes and neurons was responsible for stimulating neurite outgrowth in their stroke model, and that inhibition of miRNA machinery proteins attenuated this effect (109). Exosomal miR-17-92 (109, 110), miR-134 (105), and miR-124-3p (111) have additionally been implicated in neuroprotection. Bioengineering MSCs to produce EVs overexpressing these transcripts are currently under investigation (110, 112–114). In the majority of these studies, EVs are administered intravenously to the periphery which is important as MSC-EVs have been shown to additionally modulate the systemic immune response following traumatic CNS injuries. In a model of SCI, improvements in locomotor function have been attributed to suppression of the systemic immune response by stimulated MSC-EVs, as circulating neutrophils were reduced and monocytes were retained in the spleen (100). MSC-EVs have been shown to localize to this organ (106), and splenectomies improve neurological outcomes in models of SCI (22); it would be of interest to investigate the effect of MSC-EVs in injury models with splenectomy to determine if their beneficial effect remains.

## Conclusions

**Figure 2:**
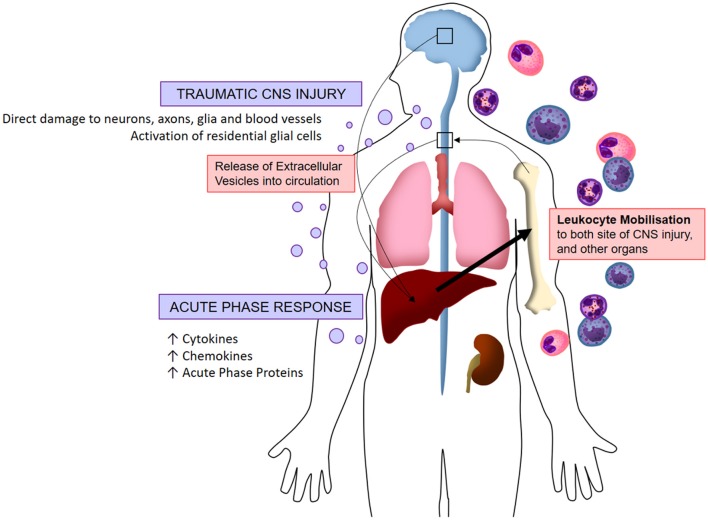
Visualized hypothesis of EV-mediated systemic inflammation response to traumatic CNS injury. Acute traumatic injuries to the brain and spinal cord induce the release of extracellular vesicles into circulation. These EVs localize to peripheral organs whereby they induce the production of pro-inflammatory molecules (chemokines, cytokines, acute phase proteins), in turn stimulating the mobilization of leukocytes which infiltrate both the CNS and peripheral organs. This systemic immune response is referred to as the acute phase response.

In the last decade, interest in EVs has increased exponentially for both biomarker and therapeutic purposes, as more studies identify EV signaling as a key component of normal physiology and pathology. However, whilst fields such as gynecology have led the way, the investigation of the role that EVs play in the context of acquired neurological diseases is relatively new. Here, we have discussed how it has been consistently shown that the circulating EV population is altered by trauma to the CNS (Figure 2). The collected evidence presented here suggests that EVs mediate the systemic response following CNS injury, and that manipulation of this pathway can protect the CNS from secondary damage. However, our understanding of the underlying mechanisms and the consequences of manipulation of the EV population, is limited, and fundamental questions remain. For instance, it is unclear whether EV biogenesis after injury is different from the mechanisms that govern basal EV production. It also remains unclear whether the absolute number of EVs in the circulation is the most important factor, or whether the enrichment of circulating EVs from CNS-derived populations, that is barely detectable in the periphery without specific markers, is more important. Moving forward, it is clear that the role of EVs in the pathogenesis of systemic inflammation following CNS injury warrants further investigation to underpin development of successful therapeutic strategies and improve functional outcomes.

## Author Contributions

Manuscript was written by AY. Manuscript was edited by DA, MR, and YC.

### Conflict of Interest

The authors declare that the research was conducted in the absence of any commercial or financial relationships that could be construed as a potential conflict of interest.
